# Brain-derived neurotrophic factor Val66Met polymorphism modulates the effects of circadian desynchronization on activity and sleep in male mice

**DOI:** 10.3389/fnins.2022.1013673

**Published:** 2023-01-09

**Authors:** Derrick J. Phillips, Scott Blaine, Naomi K. Wallace, Ilia N. Karatsoreos

**Affiliations:** ^1^WWAMI Medical Education Program, University of Idaho, Moscow, ID, United States; ^2^Department of Integrative Physiology and Neuroscience, Washington State University, Pullman, WA, United States; ^3^Neuroscience and Behavior Program, Department of Psychological and Brain Sciences, University of Massachusetts Amherst, Amherst, MA, United States

**Keywords:** neuroscience, EEG, polymorphism, sleep–wake cycle, circadian

## Abstract

**Introduction:**

Understanding how environmental interact challenges with genetic predispositions modulate health and wellbeing is an important area of biomedical research. Circadian rhythms play an important role in coordinating the multitude of cellular and tissue processes that organisms use to predict and adapt to regular changes in the environment, and robust circadian rhythms contribute to optimal physiological and behavioral responses to challenge. However, artificial lighting and modern round-the-clock lifestyles can disrupt the circadian system, leading to desynchronization of clocks throughout the brain and body. When coupled with genetic predispositions, circadian desynchronization may compound negative outcomes. Polymorphisms in the brain-derived neurotrophic (BDNF) gene contribute to variations in neurobehavioral responses in humans, including impacts on sleep, with the common Val66Met polymorphism linked to several negative outcomes.

**Methods:**

We explored how the Val66Met polymorphism modulates the response to environmental circadian desynchronization (ECD) in a mouse model. ECD was induced by housing adult male mice in a 20 h light-dark cycle (LD10:10; 10 h light, 10 h dark). Sleep and circadian activity were recorded in homozygous (Met) mice and their wild-type (Val) littermates in a standard 24 h LD cycle (LD12:12), then again after 20, 40, and 60 days of ECD.

**Results:**

We found ECD significantly affected the sleep/wake timing in Val mice, however, Met mice maintained appropriate sleep timing after 20 days ECD, but not after 40 and 60 days of ECD. In addition, the rise in delta power at lights on was absent in Val mice but was maintained in Met mice. To elucidate the circadian and homeostatic contribution to disrupted sleep, mice were sleep deprived by gentle handling in LD12:12 and after 20 days in ECD. Following 6 h of sleep deprivation delta power was increased for both Val and Met mice in LD12:12 and ECD conditions. However, the time constant was significantly longer in the Val mice during ECD compared to LD12:12, suggesting a functioning but altered sleep homeostat.

**Discussion:**

These data suggest the Val66Met mutation is associated with an ability to resist the effects of LD10:10, which may result in carriers suffering fewer negative impacts of ECD.

## Introduction

Circadian (daily) rhythms in behavior and physiology are present in nearly all plants and animals, and cycle approximately every 24 h (Moore-Ede et al., 1982). In mammals, the central circadian pacemaker is located in the hypothalamic suprachiasmatic nucleus (SCN), which synchronizes the timing of peripheral oscillators to the external lighting environment (Moore and Eichler, 1972; Stephan and Zucker, 1972; Ralph et al., 1990; Silver et al., 1990; LeSauter et al., 1996). Synchronized timing is essential for optimized brain-body systems, and disrupting this timing can lead to deleterious mental and physical health (Fishbein et al., 2021; McEwen and Karatsoreos, 2022). These effects have been reviewed elsewhere, and include impacts on metabolism (Panda, 2016), immune function (Curtis et al., 2014), and neurobehavioral function (Goel et al., 2013; Karatsoreos, 2014). Circadian desynchronization has become ubiquitous in our modern society due to nighttime lighting, shift work, and social mores like “social jet lag” (Roenneberg et al., 2012). Light at night is an emergent issue and as of 2012 is acknowledged by the American Medical Association as a significant health risk (Stevens et al., 2013). Thus, it is important to understand how altered light/dark cycles disrupt normal body cycles, and their interactions with other pre-existing conditions or genetic predispositions.

One of the most salient outputs of the circadian system, especially when disrupted, is the timing of the sleep/wake cycle. The two-process model of sleep speculates that the interaction between a wake dependent homeostatic process (process S) and the circadian process (process C) determines the timing of sleep and wakefulness (Borbély, 1982). The SCN plays a crucial role in the timing and consolidation of the sleep/wake cycle, with the drive for arousal increasing during the subjective day (Moore, 2007). Conversely, the homeostatic signal drives the need for sleep during sustained wakefulness and dissipates during non-rapid eye movement (NREM) sleep. The duration of prior wakefulness can increase NREM sleep and EEG delta power (0.5–4 Hz) and can be used as markers of the homeostatic process (Dijk et al., 1987; Franken and Dijk, 2009). While homeostatic regulation of sleep can be observed in arrhythmic SCN lesioned animals, rhythmic timing of sleep is lost (Mistlberger et al., 1983; Tobler et al., 1983; Trachsel et al., 1992) suggesting the circadian and homeostatic process interact to determine the timing, duration, and quality of sleep and wakefulness (Franken and Dijk, 2009).

Our previous studies showed that environmental circadian desynchronization (ECD) using an LD10:10 cycle causes mice to fail to entrain to the LD cycle, and disrupts the timing of many behavioral and physiological variables (Karatsoreos et al., 2011). This form of ECD also leads to misaligned sleep timing independent of sleep deprivation, decreased NREM delta power (Phillips et al., 2015), dysregulated immune function (Phillips et al., 2015; Pearson et al., 2020), metabolic dysregulation (Karatsoreos et al., 2011), and significant atrophy in prefrontal cortex neurons (Karatsoreos et al., 2011). As such, this model can be a powerful tool to explore the impact of circadian desynchronization on neurobehavioral function and sleep.

A current hypothesis for a function of sleep posits that synaptic potentiation occurs during wakefulness and is balanced by synaptic downscaling during sleep (Tononi and Cirelli, 2012, 2014, 2020). These changes in synaptic potentiation are thought to be related to changes in slow wave activity during sleep (Tononi and Cirelli, 2006; Esser et al., 2007), and can be disrupted by sleep loss and stress (Gronli et al., 2013). Many of the molecular substances that participate in sleep regulation are also regulators of synaptic plasticity (Krueger et al., 2001). Brain-derived neurotrophic factor (BDNF) is an established mediator of activity-dependent synaptic plasticity (Korte et al., 1995; Genoud et al., 2004; Bramham and Messaoudi, 2005; Gottmann et al., 2009; Murray and Holmes, 2011), is rhythmic within the SCN (Liang et al., 1998), and heterozygous BDNF deficient mice show damped SCN rhythmicity, which contributes to decreased light-induced phase shifts (Liang et al., 2000). When injected into the cortex, BDNF enhances slow wave sleep (SWS) (Kushikata et al., 1999) and delta power (Faraguna et al., 2008) and BDNF mRNA increases following sleep deprivation (Cirelli and Tononi, 2000; Taishi et al., 2001). More recent mechanistic work also clearly demonstrates that BDNF and signaling through the TrkB receptor, particularly the truncated Trb.T1 receptor, is important in sleep (Watson et al., 2015; Muheim et al., 2022). Given the role of BDNF as a modulator of synaptic plasticity and sleep, it is an ideal target toward understanding the mechanisms by which circadian desynchronization alters sleep and neural architecture.

In humans, a single nucleotide polymorphism in the BDNF gene produces an amino acid substitution of valine to methionine at codon 66 (Val66Met). This variant disrupts normal BDNF packaging into vesicles, preventing proper trafficking (Egan et al., 2003). Humans heterozygous for the Met allele show decreased hippocampal (Pezawas et al., 2004; Szeszko et al., 2005; Bueller et al., 2006) and cortical volumes (Pezawas et al., 2004), impaired hippocampal-dependent memory (Egan et al., 2003; Hariri et al., 2003), increased anxiety-like behaviors (Jiang et al., 2005; Lang et al., 2005), decreased Delta power during slow wave sleep (Bachmann et al., 2012), and impaired cognitive flexibility during sleep deprivation measured by the Stroop task (Grant et al., 2018). Studies using transgenic mice carrying the human BDNF variant describe a decrease in hippocampal volume and hippocampal-dependent memory, increased anxiety-like behaviors (Chen et al., 2006), and a decrease in dendritic length and mature dendritic spines in the prefrontal cortex (Liu et al., 2012). Given the importance of BDNF within the circadian timing and sleep systems, we hypothesized that Val66Met mice with the Met mutation (Met) would show impairment in maintaining circadian rhythms in body temperature and activity earlier than their littermate control (Val) siblings. In addition, given that we previously observed changes in delta power following ECD (Phillips et al., 2015), we hypothesized that Met mice would show increased sleep fragmentation and decreased NREM delta power earlier than littermate Val mice.

## Materials and methods

### Animals

We received C57BL/6 BDNF Val66Met knock-in mice from Francis Lee (Cornell University) and their generation was previously described (Chen et al., 2006). Heterozygous Met/Val mice were maintained on the C57BL/6 background, and the resulting male Val/Val (control), and Met/Met mice were used. Before experiments, all mice were raised in normal lighting conditions (12 h on, 12 h off light/dark cycle; LD12:12) with food and water available *ad libitum*. Adult mice (4–6 months old) were used for all surgical procedures. One week prior to surgical procedures, mice were moved from the breeding colony and placed into sound attenuated and ventilated isolation cabinets where light at cage level was maintained ∼200 lux using white LEDs. All experimental procedures were approved by the Washington State University Animal Care and Use Committee.

### Surgical procedures

Adult male mice (Val/Val *n* = 12, and Met/Met *n* = 11) were initially anesthetized using 5% isoflurane in oxygen, and then maintained at 2–2.5% isoflurane during surgical procedures. First, body temperature and activity telemeters (VitalView/E-Mitter, Starr Life Sciences, Oakmont, PA) were implanted into the peritoneal cavity. Next, electroencephalogram (EEG) and electromyogram (EMG) electrodes were implanted as previously described (Clegern et al., 2012). Briefly, the skull surface was exposed and two stainless-steel screws (Antrin Miniature Specialties, Inc., Fallbrook, CA) were placed over the frontal lobe to measure EEG (0.5 mm lateral to the midline, 1 mm anterior to bregma), and two over the parietal lobe (1.5 mm lateral to the midline, 1.5 mm anterior to lambda) as a ground and a reference. Two additional screws implanted in the parietal lobe were used as additional anchors for the head stage. These electrodes were then soldered to a PCB head mount with a 6-pin connector (Pinnacle Technology, Inc., Lawrence, KS). Attached to the PCB board, two braided stainless-steel wires with ∼2 mm insulation removed at the terminating end were inserted into the neck muscle to record EMG. The electrodes and the PCB board (with the 6-pin connector still exposed) were then enclosed in a light activated composite resin. Mice were allowed at least 1 week to recover following surgery.

### Sleep recordings and circadian desynchronization experiment

Following surgery, mice were single housed in their home cage and transferred into a 10-inch diameter circular sleep chamber during sleep recordings. The home cages and sleep chambers were placed on telemeter pads which continuously recorded body temperature and activity in 5 min bins (Vital-view/E-Mitter). For baseline sleep recordings (LD12:12), mice were tethered through a commutator to the sleep system (Pinnacle Technology, Inc.) and data were recorded with a 2 kHz sample rate, 100x gain, and filtered with a high pass of 0.5 Hz, and low pass of 120 Hz. Mice were allowed to acclimate to the sleep recording setup 5 days before baseline sleep was recorded for 24 h. Mice were then returned to their home cages, and the light/dark cycle was altered to a 10 h on, 10 h off cycle (LD10:10) to induce environmental circadian desynchronization (ECD), as we have previously described (Karatsoreos et al., 2011; Phillips et al., 2015). After 20, 40, and 60 days of ECD, mice were transferred back into the sleep recording chambers for 5 days to determine changes in sleep patterns due to ECD.

In a second cohort of animals, we followed the same surgical procedures and initial experimental setup stated above. Following a baseline sleep recording, mice were subjected to sleep deprivation by gentle handling. Mice were carefully observed, and if they started to show signs of sleep, they were gently prodded using a fine haired paintbrush over 6 h starting at light onset. Following deprivation, mice were given 24 h of recovery sleep. Afterward, mice were returned to their home cages and placed into our ECD protocol for 20 days. After 20 days in ECD, mice returned to the sleep recording chambers for 5 days. After 72 h of this initial recording period, sleep deprivation by gentle handling started at light onset and continued for 6 h. Following sleep deprivation, mice were allowed recovery sleep for 24 h.

### Sleep scoring

Archived data were loaded into Sirenia Sleep Pro (Pinnacle Technology, Inc., Lawrence, KS) and parsed into 10 s epochs for analysis. EEG spectral power was averaged into the delta frequency bandwidth (0.5–4.0 Hz), and EMG total power (0.1–1,000 Hz) was calculated. A first pass sleep scoring was done within Sirenia using a cluster technique based on the density clusters from the EEG delta power and total EMG power. Following cluster scoring, visual confirmation of state was done and corrections were made where necessary.

### Sleep data analysis

Scored sleep data and the corresponding EEG delta power were imported into MATLAB (R2015a, MathWorks, Natick, MA) for further analysis. For our analysis, we defined a day as one cycle of the light and dark period. The control condition was defined as a 24 h day, with 12 h light period and a 12 h dark period. With our sleep scoring analysis binning data into 10 s epochs, each hour for the control condition consisted of 60 min, or 360 epochs. For circadian disruption a day was defined as one cycle of light/dark. Each ECD day was 20 earth hours with 10 h of light and 10 h of dark. For our analysis, each ECD day was divided into 24 segments. Each hour consisted of 50 min, or 300 epochs. To account for the differences in epochs between the control and ECD conditions we calculated the percent time in wake/sleep states.

Wake to sleep transitions were calculated over a recording day, then normalized to the time difference between the baseline and disrupted recording day, and reported as the number of transitions per hour. A transition from wake to sleep was defined as a wake state lasting at least 30 s before entering NREM sleep. Delta power and scoring data from the sleep recordings were parsed into 1 h bins. Statistical comparisons made are described below.

### Circadian data analysis

Locomotor activity was recorded continuously over the entire duration of the experiment. Activity counts were exported in 5 min bins and imported into the ImageJ plugin ActogramJ (Schmid et al., 2011). Chi-squared periodograms were calculated from 5 days corresponding to the animals being in the sleep chambers. To measure the robustness of the circadian rhythm power, chi-squared periodograms were generated to compare the 7 day rhythmicity between the baseline and following 20 days ECD. The statistical chi-squared Qp value was calculated, and the 20 and 24 h values were compared between baseline and 20 days ECD.

### Statistics

Statistical analyses were undertaken using Prism 7 (GraphPad Software, Inc., La Jolla, CA). Two-tailed *t*-tests, one-way ANOVA and two-way ANOVA with Sidak’s multiple comparisons test were run where appropriate. Multiple comparisons were done using Tukey test. Comparison of fit between a linear vs. exponential decay model were done using Akaike’s Informative Criteria, to select the model most likely to have generated the data. Exponential decay data were fit using the following formula Y = (Y0–Plateau) × exp (−K × X) + Plateau, where X is time, Y is the start of the decay, the plateau is the lower asymptote, and K is the rate constant, where the time constant tau is 1/K. When the preferred model from the grouped data was an exponential decay, the tau from each mouse was calculated.

## Results

### Sleep timing

**FIGURE 1:**
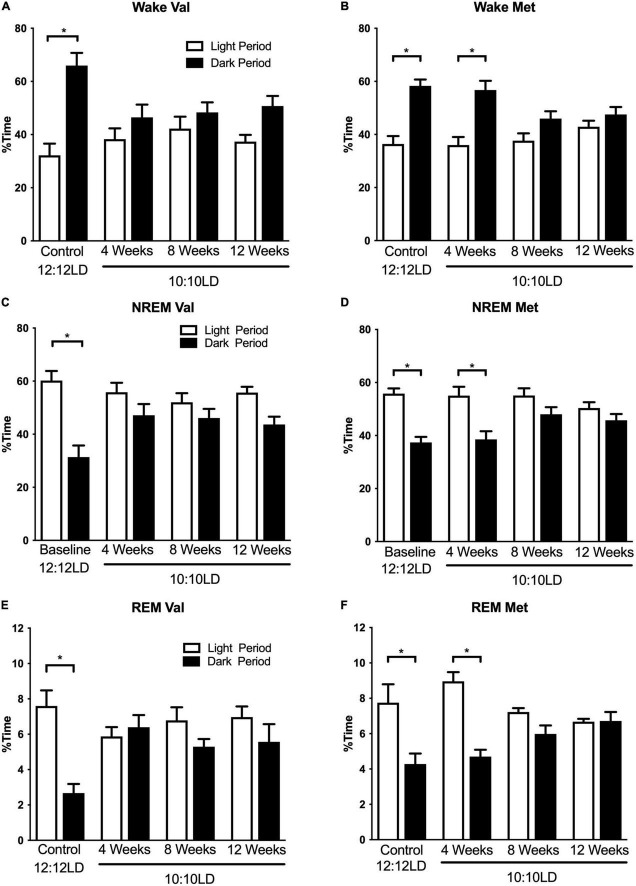
Environmental circadian desynchronization alters distribution of wake and sleep across the light and dark period differently in Val and Met animals. Graphs demonstrate changes in niche appropriate sleep timing under baseline and ECD conditions. In baseline conditions, Val mice **(A)** show a significant difference in wake time between the light (white bars) and dark (black bars) phase. However, these differences were no longer significant following 20, 40, and 60 days of LD10:10 ECD. Met mice **(B)** show a similar light/dark difference during the baseline recording. Following 20 days of LD10:10 ECD, light light/dark difference wake time is maintained, but not after 40 and 60 days of LD10:10 ECD. These differences are also present during NREM sleep for the Val **(C)**, and Met **(D)**, and REM sleep **(E)** and **(F)**. Two-way ANOVA; *p < 0.05.

Environmental circadian desynchronization had a significant effect on the time-of-day differences in sleep and wake timing. There was a main effect of circadian desynchronization on wake timing in both Val and Met mice [Figures 1A,B; Two-way ANOVA, Val *F*_(1,40)_ = 27.46, *p* < 0.001; Met *F*_(1,24)_ = 48.31, *p* < 0.001], however, there was a significant interaction [Val *F*_(3,40)_ = 4.65, *p* = 0.007; Met *F*_(3,24)_ = 4.77, *p* = 0.0096]. *Post hoc* analyses revealed significant time-of-day differences in wake time for both Val and Met mice in LD12:12 (Val, *p* < 0.001; Met, *p* < 0.001), with more wake time during the dark period in both groups. This relationship was no longer significant in the Val group after 20 days (*p* = 0.53), 40 days (*p* = 0.76), and 60 days (*p* = 0.11) of LD10:10. In Met mice, the difference in wake time was maintained after 20 days of ECD (*p* < 0.001) but was lost after 40 days (*p* = 0.18), and 60 days (*p* = 0.70) of LD10:10.

Environmental circadian desynchronization using LD10:10 also resulted in significant time of day differences in NREM sleep timing for both Val and Met mice [Figures 1C,D; Two-way ANOVA, Val *F*_(1,40)_ = 30.95, *p* < 0.001; Met *F*_(1,24)_ = 40.45, *p* < 0.001], however, there was a significant interaction [Val *F*_(3,40)_ = 4.28 *p* = 0.01; Met *F*_(3,24)_ = 3.46, *p* = 0.03]. *Post hoc* analysis revealed a statistical difference in NREM sleep timing between the light and dark period during baseline recording in both Val and Met mice, with more NREM sleep during the light period (Val, *p* < 0.001; Met, *p* < 0.001). This difference was no longer statistically significant for the Val group after 20 days (*p* = 0.31), 40 days (*p* = 0.67), and 60 days (*p* = 0.08) of LD10:10. The Met group maintained a time-of-day difference in NREM sleep timing after 20 days of LD10:10 (*p* < 0.001), but was lost after 40 days (*p* = 0.24), and 60 days (*p* = 0.62).

Rapid eye movement sleep timing was also significantly affected by LD10:10 induced ECD for both Val and Met mice (Figures 1E, F; Two-way ANOVA, Val *F*_(1,40)_ = 7.58, *p* = 0.009; Met *F*_(1,24)_ = 33.07, *p* < 0.001), with a significant interaction (Val *F*_(3,40)_ = 4.88, *p* = 0.06; Met *F*_(3,24)_ = 6.52, *p* = 0.002). Like the wake and NREM findings, *post hoc* analysis revealed a significant difference in REM sleep timing between the light and dark period during the baseline recording for both groups (Val *p* < 0.001; Met *p* < 0.001), with more time spent in REM sleep during the light period. This relationship was no longer significant for the Val group after 20 days (*p* = 0.98), 40 days (*p* = 0.99), and 60 days (*p* = 0.61) of LD10:10. The Met group maintained niche appropriate REM sleep timing after 20 days (*p* < 0.001) of LD10:10, but this was lost after 40 days (*p* = 41) and 60 days (*p* > 0.99).

### Sleep transitions

**FIGURE 2:**
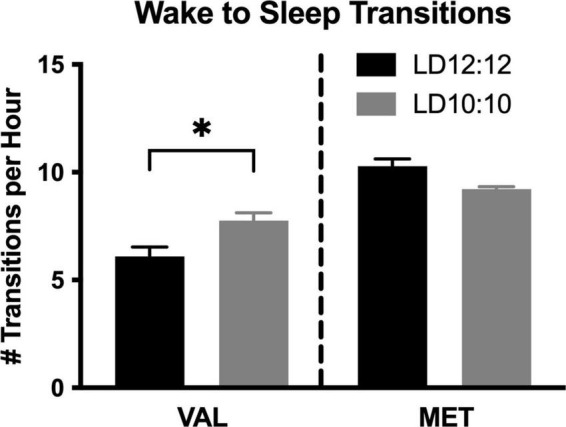
Wake to sleep transitions are differentially impacted by ECD in Val and Met mice. Graphs show changes in wake to sleep transitions. LD10:10 ECD (20 days) resulted in an increase in the number of transitions from wake to sleep in the Val mice (black bar), but no difference was found in the Met mice (gray bar). One-Way ANOVA; *p < 0.05.

Environmental circadian desynchronization led to an increase in the number of wake to sleep transitions per hour in Val mice, but not Met mice. There was a significant difference in the number of wake to sleep transitions [Figure 2; One-way ANOVA, *F*_(3,14)_ = 26.53, *p* < 0.001]. *Post hoc* analysis showed a significant difference between Val and Met in the number of wake to sleep transitions during LD12:12 (*p* < 0.001), with Met mice having more transitions. After 20 d of LD10:10 ECD, Met mice continued to have more transitions than Val mice (*p* = 0.049). Val mice had an increase in the number of transitions following 20 days of LD10:10 ECD compared to LD12:20 (*p* = 0.016), while the number of transitions from Met mice remained unchanged (*p* = 0.23).

### Delta power

**FIGURE 3:**
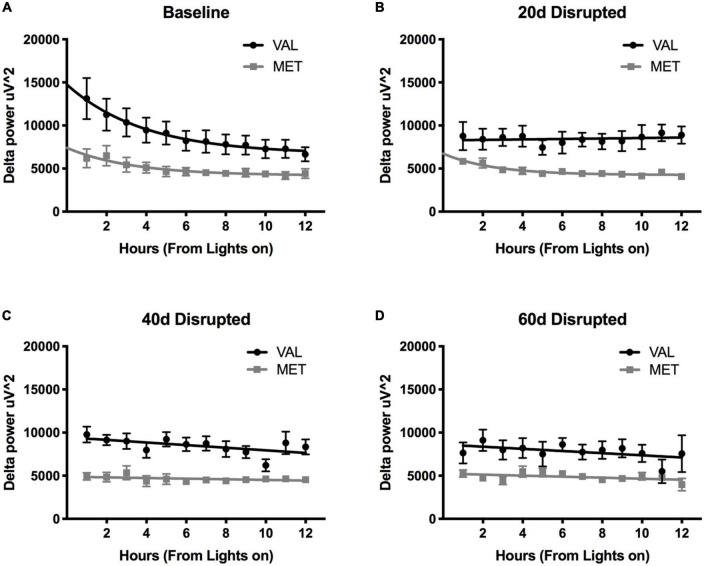
Length of time in ECD changes delta power differently in Val and Met mice. Graphs depict changes in NREM sleep delta power (0.5–4 Hz) in Val (black lines) and Met (gray lines) starting at light onset for baseline LD12:12 **(A)**, 20 days **(B)**, 40 days **(C)**, and 60 days **(D)** of LD10:10 ECD. Best fit lines were generated by comparing an exponential decay to a straight line. Light onset NREM delta power from baseline recordings **(A)** best fit to an exponential decay model, with increased delta power at light onset and dissipates over time. However, after 20 days **(B)**, 40 days **(C)**, and 60 days **(D)** of LD10:10 ECD, delta power from the Val mice best fits a linear model, showing no rise in light onset delta power. After 20 days of LD10:10 ECD, Met mice still fit an exponential decay in light onset delta power, but best fit a liner model after 40 days or 60 days.

During LD12:12 baseline and LD10:10 ECD, there was a significant main effect of genotype on delta power during NREM sleep [Figure 3; Two-way ANOVA, LD12:12 *F*_(1,83)_ = 63.04, *p* < 0.0001; 20 days *F*_(1,83)_ = 98.14, *p* < 0.0001; 40 days *F*_(1,82)_ = 152.0, *p* < 0.0001; and 60 days *F*_(1,80)_ = 47.43, *p* < 0.0001], with Val mice having significantly higher delta power compared to Met mice. There was also a main effect on time between the Val LD12:12 and Val after 40 days of desynchronization [Two-Way ANOVA, *F*_(11,95)_ = 2.33, *p* = 0.0141], and between Met LD12:12 and Met after 20 days desynchronization [Two-Way ANOVA, *F*_(11,71)_ = 2.94, *p* = 0.0029]. The most striking difference was a flattening of light onset delta power during the desynchronization. When comparing a linear fit to exponential decay, the baseline recordings for both Val LD12:12 and Met LD12:12 best fit an exponential decay. Following 20, 40, and 60 days of CD, the Val mice delta power best fit a linear model. The Met mice light onset delta power still fit the exponential decay model after 20 days of LD10:10 [Difference in Akaike information criterion with a correction for sample sizes (AICc), 0.39, 0.73, and 1, respectively], but best fit a linear model following 40 and 60 days of CD. Looking at the time constant for the decay, the tau values were not statistically significant between the Val LD12:12, Met LD12:12, and Met LD10:10 for 20 days (One-way ANOVA, *F* = 0.469, *p* = 0.22).

### Sleep deprivation

**FIGURE 4:**
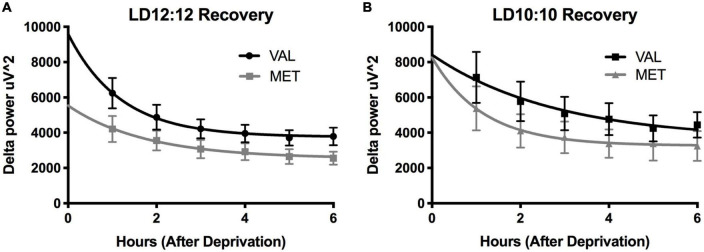
Environmental circadian desynchronization does not significantly impact the EEG response to sleep deprivation in either genotype. Graphs depict changes in EEG responses following sleep deprivation. Sleep deprivation significantly increased delta power during NREM recovery sleep. Val (black) and Met (gray) mice in baseline LD12:12 **(A)** show an increase in NREM delta power following 6 h of sleep deprivation that decays over time. After 20 days of LD10:10 ECD **(B)**, the same relationship can be observed in both groups during recovery sleep following 6 h of sleep deprivation.

**FIGURE 5:**
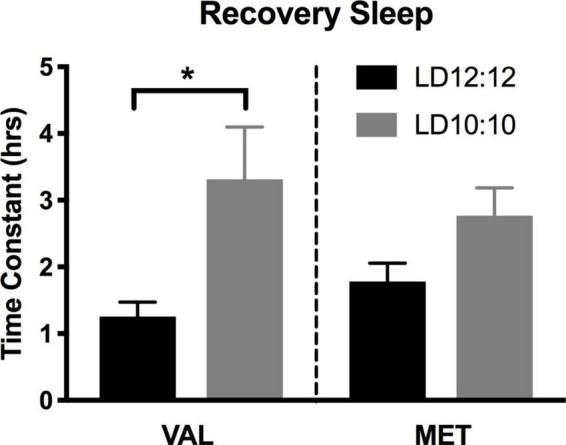
Time constant of recovery sleep in Met mice is different than in Val mice. Graphs depict the time constant (tau) during recovery sleep. In Val mice, tau during recovery sleep was significantly longer after 20 days of LD10:10 ECD when compared to the LD12:12 recovery period. No statistically significant difference in tau was found in the Met mice. One-way ANOVA; *p < 0.05.

Light onset NREM delta power during baseline in LD12:12 was significantly higher for Val mice compared to Met mice, similar to our results above [Two-way ANOVA, *F*_(1,119)_ = 14.99, *p* = 0.0002]. After 20 days in LD10:10 ECD, there was a significant effect of genotype on delta power, with Val mice showing flattened delta power at light onset, while Met mice continue to show the expected delta power change at lights on [Two-way ANOVA, *F*_(1,115)_ = 6.07, *p* = 0.015]. For both Val and Met mice, a 6 h sleep deprivation in LD12:12 increased NREM delta power during the recovery period, with a main effect of time and genotype [Figure 4A, Two-way ANOVA, Time, *F*_(5,60)_ = 3.86, *p* = 0.004; Genotype, *F*_(1,60)_ = 15.65, *p* = 0.0002], with no interaction. After 20 days in LD10:10, a 6 h sleep deprivation increased NREM delta power in both Val and Met groups, with a main effect of genotype [Figure 4B, Two-way ANOVA, *F*_(1,54)_ = 5.64, *p* = 0.02]. From the recovery data, exponential decay of NREM delta power was calculated for each animal and the tau values were compared between groups. There was a significant difference in the tau values between the groups (Figure 5, One-way ANOVA, *F* = 3.8, *p* = 0.027). Multiple comparisons revealed a significant difference between the Val LD12:12, and Val LD10:10 (*p* = 0.03), with Val LD10:10 having a longer tau than Val LD12:12.

### Circadian rhythmicity

**FIGURE 6:**
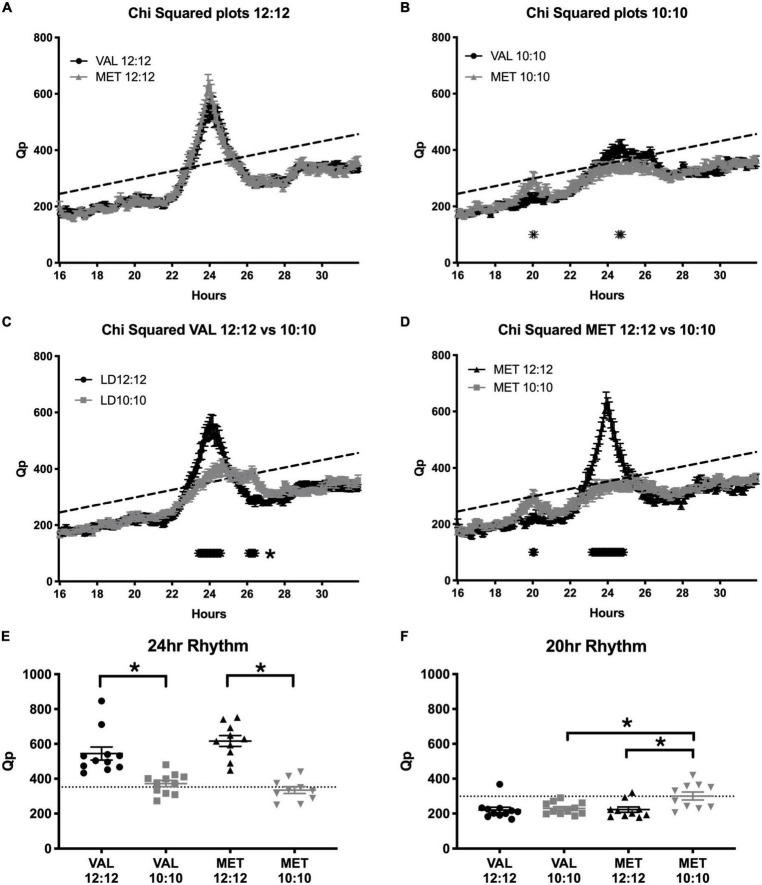
Met mice show a stronger period adaptation to ECD than do Val mice. Graphs show Chi-squared values during baseline LD12:12 conditions and after 20 days in LD10:10 ECD. During the baseline recording **(A)**, there was a robust locomotor rhythm centered on 24 h in both Val (black line) and Met (gray line) animals. After 20 days in ECD **(B)**, Met mice had a higher Qp value around a 20 h rhythm, and Val mice had a higher Qp value around a 24 h rhythm. When comparing rhythms between the control (LD12:12; black line) and ECD (LD10:10; gray line) cycles, Val mice **(C)** had a less robust rhythm at 24 h, and had a longer period around 26 h. Met mice **(D)** had a higher Qp value around a 20 h period in a LD10:10 cycle, and a blunted 24 h rhythm. Qp values from a 24 h rhythm **(E)** were above the significance line for all Val and Met mice in a LD12:12 cycle, while only 7/11 Val mice and 3/10 Met mice were above the significance line. Qp values from a 20 h rhythm **(F)** were above the significance line in 1/11 Val mice and no Met mice in a baseline conditions, while 1/11 Val mice and 5/10 Met mice were above the significance line in ECD. *p < 0.05.

During the baseline LD12:12 recording, there was a robust rhythm, where the critical values of Qp reached a statistical threshold centered on a 24 h rhythm in both Val and Met animals (Figure 6A, Val = 544.2 ± 37.5; Met = 616.6 ± 31.6; *p* = 0.99; Statistical *Qp* = 352.3). After 20 days in LD10:10 ECD, there were significant differences between the groups centered at 20 and 24 h. Met mice had a higher Qp value around a 20 h rhythm than Val mice (Figure 6B, Val = 229.4 ± 10.5; Met = 300.8 ± 22.9; *p* = 0.02; Statistical *Qp* = 298.9). This 20 h rhythm was not present in Met mice under the LD12:12 cycle but appeared during the LD10:10 cycle (Figure 6D, Met LD12:12 = 222.6 ± 15.2; LD10:10 = 300.8 ± 22.9, *p* = 0.01). Val mice did not show a 20 h component in either LD12:12 or LD10:10 (Figure 6C, Val LD12:12 = 220.6 ± 16; LD10:10 229.4 ± 10.5). The 24 h rhythm in LD10:10 was significantly reduced for both Val (LD12:12 544.2 ± 37.5; LD10:10 372.3 ± 18.0; *p* < 0.01) and Met (LD12:12 = 616.6 ± 31.6; LD10:10 = 334.9 ± 19.9 *p* < 0.01) mice, but no difference was found between the genotypes. Qp values from a 24 h rhythm (Figure 6E) were above the significance line for all Val and Met mice in a LD12:12 cycle, while only 7/11 Val mice and 3/10 Met mice were above the significance line under LD10:10. Qp values with a 20 h rhythm (Figure 6F) were above the significance line in 1/11 Val mice and no Met mice in a LD12:12 cycle, while 1/11 Val mice and 5/10 Met mice were above the significance line in a LD10:10 cycle.

## Discussion

In humans, the Val66Met polymorphism is associated with decreased hippocampal (Pezawas et al., 2004; Szeszko et al., 2005; Bueller et al., 2006) and cortical volume (Pezawas et al., 2004), impaired hippocampal-dependent memory (Egan et al., 2003; Hariri et al., 2003), increased anxiety-like behaviors (Jiang et al., 2005; Lang et al., 2005), and increased vulnerability to the effects of sleep deprivation on cognitive tasks (Grant et al., 2018). This would suggest some distinct evolutionary disadvantages, yet the polymorphism persists. Intriguingly, our results in mice suggest Met carriers are more capable of adapting to extreme changes in LD cycle. We conjecture that it is possible that Met/Met carries have a more “adaptable” circadian timing system, or a more flexible relationship between Process C and Process S. It is possible this increased adaptability may provide an environmental advantage and thus could provide one reason for the persistence of the polymorphism. It is important to consider that a genetic predisposition that may be a disadvantage in one context, might provide an advantage in another.

Wild-type Val mice housed in LD10:10 were unable to entrain to a 20 h day, showed decreased periodicity around 24 h, lost niche appropriate sleep timing, and had flattened light onset delta power. However, mice carrying the Val66Met (Met) polymorphism were more likely to be rhythmic in a 20 h day, maintained appropriate sleep timing, and retained a normal light onset delta power response, at least up to 20 days in LD10:10. After sleep deprivation, both groups had similar increases in delta power during NREM sleep in both LD12:12 and LD10:10. This finding is interesting considering that while mice carrying the Met allele have increased stress induced anxiety-like behaviors (Chen et al., 2006), they appear to be more resistant to the effects of ECD on sleep timing and delta power. Thus, if one considers ECD a form of “stress,” then not all stressors are created equal or have the same interaction with the Val66Met polymorphism.

If the circadian timing system is not functioning properly, behavioral and physiological processes can be compromised, reducing resilience to challenges in the environment. Indeed, both REM sleep changes and peripheral measures of BDNF seem to be predictive of resilience in the face of chronic stressors (Sweeten et al., 2020). The SCN clock is an important determinant of the circadian timing of sleep, and SCN lesions result in a loss of consolidated sleep without changes in total sleep time (Mistlberger et al., 1983; Tobler et al., 1983). Thus, disrupting the SCN with an irregular light cycle (such as LD10:10) may lead to a loss of consolidated sleep. BDNF may also play a key role in sleep timing by regulating how the SCN responds to altered LD cycles, as BDNF is rhythmically expressed in the SCN (Liang et al., 1998), and mice deficient in the BDNF receptor TrkB have a diminished phase shift response to light (Allen et al., 2005). Our present results show that Val mice lose niche appropriate timing for sleep sooner than their Met littermates. There are several potential explanations for these findings. The BDNF Met mutation leads to a decrease in prefrontal neuron dendrite complexity (Liu et al., 2012). It is possible the mutation also leads to a weakening in synaptic strength of retinohypothalamic tract fibers terminating on the SCN in Met mice, which would reduce photic input to the SCN, and hence lead to less effects of the altered light-dark cycle. An additional potential mechanism could be a weakening in synaptic connectivity within the SCN itself, which might lead to an SCN network that is less robust, and hence more adaptable to non-24 h periods. Previous studies show mice lacking vasopressin receptors are more responsive to extreme changes in light cycle due to less rigid coupling between SCN cells (Yamaguchi et al., 2003). The Met polymorphism may also lead to weaker coupling of SCN cells, which serves to improve the ability of the mouse to synchronize to exotic light-dark cycles. Finally, a decrease in synaptic strength on SCN outputs, or in SCN targets, caused by the Val66Met polymorphism may reduce the overall effect of the photic stimuli on downstream SCN targets. At this stage, it is too early to know which (or all) of these outcomes contribute to the observed effects. However, a chronic phase advance circadian disruption model seems to reduce BDNF levels in the hippocampus (Chen et al., 2021), suggesting that links between chronic circadian rhythm desynchronization and BDNF certainly extend beyond the SCN.

In addition to sleep timing changes, our results show ECD influences NREM sleep intensity as measured by delta power thereby confirming our previous work (Phillips et al., 2015). NREM sleep delta power is thought to reflect the homeostatic process, with prolonged wakefulness leading to an increase in subsequent sleep intensity (Franken et al., 1991). Using an exponential decay model where appropriate, we measured the time constant tau to assess delta power dissipation. After 20 days of LD10:10 ECD, Val mice have a flattened light onset delta power, which persists for at least 60 days. Similar to the sleep timing data, Met mice maintained robust sleep onset delta power decay after 20 days of LD10:10 ECD, but have a flattened delta power after 40 days, which persists for at least 60 days. Met mice maintain an exponential decay in delta power which suggests the homeostatic drive for sleep is still intact. This pattern of results suggests that Met mice may be more resistant to the effects of ECD.

After 6 h of sleep deprivation, both Val and Met showed an increase in recovery sleep delta power during baseline LD12:12, and after 20 days of LD10:10 ECD. These data suggest that the homeostatic drive for sleep is intact during ECD, and the disruption of the circadian process may be leading to the inappropriate timing and decreased light onset delta power found in sleep. The tau values calculated from the exponential decay suggest that compared to recovery sleep in LD12:12, after 20 days of LD10:10 ECD, Val mice take longer to dissipate their sleep need during recovery sleep. While the homeostatic drive for sleep during ECD is still intact, it appears to be more difficult to dissipate sleep need during ECD. This difference was not observed in Met mice. This may indicate differences in the efficiency of synaptic downscaling following ECD, and that Met mice seem not to show this same reduction in efficiency. It is possible that Met mice represent a “floor effect,” but considering that under baseline LD12:12 conditions there is no difference in the tau of decay between genotypes, a different mechanism may be involved. Future experiments could explore the long-term impacts of circadian desynchronization on sleep function in these two genotypes, considering that previous work using the LD10:10 ECD model demonstrates there are some sustained changes in sleep intensity that persist after re-alignment (Hasan et al., 2018).

Similar to what has been observed in human sleep studies in these genotypes (Bachmann et al., 2012), overall delta power for baseline and during ECD was lower in Met mice than Val. BDNF, in addition to being a potentiator of synaptic plasticity, plays a role in sleep, as it increases NREM sleep time and slow wave activity (SWA) (Kushikata et al., 1999; Faraguna et al., 2008). In humans heterozygous for the Met allele, cortical volume is reduced (Pezawas et al., 2004). Since delta power is generated by synchronous neural activity (Amzica and Steriade, 1995; Steriade et al., 2001; Vyazovskiy et al., 2007, 2009), a loss in cortical volume or connectivity could result in the lower delta power during sleep seen in our Met mice. Recently, it has demonstrated that humans homozygous for Val show a stronger link between recognition memory and slow oscillation-spindle coupling than do Met carriers, and this is associated with better performance (Halonen et al., 2022).

Sleep and circadian rhythms are intimately linked, with BDNF likely playing a key role in their interaction (Rahmani et al., 2020). Our results demonstrate that a common polymorphism in the BDNF gene can have profound effects on maintaining behavioral rhythmicity, sleep timing and sleep quality. A recent study in humans suggests that Met carriers show a reduced inflammatory response to simulated shift work (as measured by IL-6) (Satterfield et al., 2020), further supporting this conclusion. Although this mutation leads to several negative cognitive effects, the ability to stay rhythmic despite changing environmental conditions may outweigh the cognitive consequences. Taken together, these results suggest that perhaps in our modern society, with the ability to constantly change light cycles, carriers of the Met allele may have advantages Val carriers do not. Understanding that genetic predispositions to particular responses are likely context dependent, should encourage us to think more broadly when ascribing “advantage” or “disadvantage” monikers to any particular genetic polymorphisms.

## Data availability statement

The raw data supporting the conclusions of this article will be made available by the authors, without undue reservation.

## Ethics statement

This animal study was reviewed and approved by the Washington State University IACUC.

## Author contributions

IK and DP conceptualized and planned the work and wrote the manuscript. DP and NW undertook the work. DP and SB analyzed the data. DP undertook statistical analyses. All authors contributed to the article and approved the submitted version.
